# Household food security and dietary diversity in south‐eastern Nigeria

**DOI:** 10.1111/mcn.13599

**Published:** 2023-12-04

**Authors:** Ijeoma C. Ukonu, Carol A. Wallace, Nicola M. Lowe

**Affiliations:** ^1^ School of Sport and Health Sciences The University of Central Lancashire Preston UK

**Keywords:** dietary diversity, food access, food choice, food insecurity, food security, household food access, Nigeria

## Abstract

The aim of this study was to investigate household food security (access) level and the dietary diversity of households in the Nsukka Local Government Area in South‐eastern Nigeria. From 20 local communities of Nsukka, 390 women were randomly sampled from the women's group and asked to complete a survey that determined the Household Food Insecurity Access Scale scores and the Household Dietary Diversity Scores (HDDS). The descriptive results indicated a high level of food insecurity with 82.6% households reporting various degrees of food insecurity. Over half of the sampled population experienced insufficient food quality. They either ate unwanted food (65.9%), limited variety (63.1%), or unpreferred food (64.6%). Some households experienced insufficient food intake by going a whole day without food (38.2%), go to sleep hungry (45.1%), or have no food of any kind (49%). The analysis of variance showed no significant difference (*p* = 0.428) in the food security level of households headed by males as compared with those headed by females. Approximately 53.6% of households fell at or below the average HDDS; males headed 48% of these households, while females headed 64%. The chi‐square test indicated factors associated with household food security including age, education, work status and income, whereas the gender of the household head, household size and marital status were not significantly associated. Public–private partnerships, nutrition orientation and food intervention programs could improve food security in this area.

## Key messages

- This study found a high level of household food insecurity in Nsukka Local Government Area (NLGA) at 82.6%, with 60.3% of households being severely food insecure and 13.3% and 9% mildly and moderately food insecure, respectively, resulting in anxiety for most households.
- The majority ate limited variety, unwanted and unpreferred food, with more households consuming starchy foods than protein‐rich foods. About 53.6% households fell at or below average dietary diversity.
- There was no difference in household food security based on the gender of the household head.
- Food security was associated with education, age, income and work status.
- Rural households within NLGA require food intervention programs to alleviate food insecurity.

## Graphical abstract

High household food insecurity (82.6%) exists in Nsukka Local Government Area. Food security was associated with education, age, income and work status. Urgent food intervention programs are required to alleviate food insecurity.

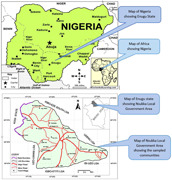

## 1 INTRODUCTION

Food security has been defined by the Food and Agricultural Organisation of the United Nations (Food Agricultural Organisation, 1996) as ‘when all people, at all times, have physical and economic access to sufficient, safe and nutritious food to meet their dietary needs and food preferences for an active and healthy life’. The four dimensions of food security describe and promote adequate food intake for health and well‐being: accessibility, availability, utilisation and sustainability (Gibson, 2012). Compromising any of these factors results in food insecurity (Bakhtsiyaraval et al., 2021).

The sustainable development goals (SDG) address poverty and hunger levels towards achieving food security and improving nutrition (Food and Agricultural Organisation, 2018). However, ‘The World is not yet on track to achieve zero hunger by 2030’ (Global Hunger Index, 2020). A report from the Food and Agricultural Organisation, (2019) shows that 2013.8 million people around the world are moderately or severely food insecure; this is an increase from 1929.6 million people in 2017 and 1801.9 million people in 2016. The United Nations (2018) and World Bank (2019) reports on Sustainable Developmental Goals and Poverty, reveal that 783 million people live in extreme poverty globally, earning $1.90 per day or less, mostly in developing regions of Asia and Africa.

Nigeria is one of the West African countries experiencing food insecurity. The Food and Agricultural Organisation (2021) estimated that 12.1 million Nigerians are in a food insecurity crisis, and it is feared to increase to 16.9 million people if humanitarian support and government interventions are not scaled up. Although endowed by nature with extensive land mass, varieties of crops in different ecological zones with optimal yield, oil wells and increasing population, harnessing these resources to provide national food sufficiency has proved problematic (Oriola, 2009). This has been exacerbated by the COVID‐19 pandemic (Food and Agricultural Organisation, 2021), climate extremes, civil insecurity (Famine Early Warning Network System, 2020), bad governance and corruption (Igbinedion & Aihie, 2015).

NLGA is the study area, geographically located between latitudes 6°30′ and 7°2′N and Longitudes 7°7′ and 7°38′E with a land area of 475 km^2^. Its indigenes are predominantly subsistent farmers, and most communities are rural (Agwu & Igbinosa, 2014). There is limited research that has investigated household food security in NLGA. However, past studies revealed a high level of poverty at 69.2% with low socioeconomic development (Ali & Agbiogwu, 2014) and 70% live below the poverty line of $1.25 per day (Ataguba et al., 2011). NLGA hosts one of Nigeria's first and most famous universities. Nzeagwu and Aleke (2016) reported that the University of Nigeria Nsukka positively influences women's education and, indirectly, their food security level. The issue of household food security and gender remains debatable (Adepoju & Adejare, 2013). Nwuba (2021) revealed that the women of Southeast Nigeria make tangible contributions to the provisions and progress of their households regardless of the patriarchal and patrilineal culture in the region, which apportions family wealth to males and the females are expected to access wealth through their husbands or brothers. The social‐cultural beliefs of the southeasterners (the Igbos) arrogate power to the man, and the feminine gender submits to him (Nduka & Ozioma, 2019). Males are characterised by their ability and ego as sole providers of the household (Dike, 2015; Ezenwanebe, 2006). Culturally, they value large households, be they nuclear or extended (Ikwubuzo, 2012), which can increase labour for agricultural productivity. The argument around household size is controversial. While large household size can negatively impact food security levels (Abu & Soom, 2016; Nzeagwu & Aleke, 2016), it could also provide labour and income earnings, especially to rural households (Okonkwo et al., 2021).

Women generally are the most affected by poverty and food insecurity. However, they play substantial roles in rural economic activities, need more access to training, economic empowerment for businesses, education and political positions, and have lower social status (Felker‐Kantor & Wood, 2012; Nigeria Millennium Development Goals, 2016). In the Southeast region, women are responsible for food preparation and distribution and greatly support one another and their husbands. A network of women's groups meets regularly at community level; these are self‐support groups, and most married women, widows, and divorcees belong to these groups. It gives them a sense of belonging and provides friendship and help for one another in distress. The society respects these groups, and it could provide a voice for the women (Nwuba, 2021).

The aim of this study is to investigate the level of household food security and dietary diversity level of households in NLGA of Enugu State in the South‐East of Nigeria. Our specific objectives were (1) to measure the prevalence of Household Food Insecurity (access) in NLGA; (2) to examine factors associated with household food security in the locality; and (3) to explore household dietary diversity. This research is vital as there is a continuing debate on the gender of household head and household food security level (Adepoju & Adejare, 2013; Felker‐Kantor & Wood, 2012; Matemilola & Elegbede, 2017; Nwaka et al., 2020) and age impact on food security (Department of Environmental, Food and Rural Affairs, 2021; Worku, 2023). This information is important to inform the policy direction for the alleviation of food insecurity in this region.

## 2 METHODS

### 2.1 Research instruments

The HFIAS (Coates et al., 2007) and the Household Dietary Diversity Score (HDDS) (Swindale & Bilinsky, 2006) were identified for data collection in this research.

The HFIAS is an experience‐based scale survey that measures household food insecurity scores and prevalence levels in the food accessibility dimension of food security. It contains nine food insecurity questions and nine occurrence questions involving a 30‐day recall of a household's food access. It investigates the three domains of HFI—anxiety and uncertainty, insufficient quality and insufficient food intake and its physical consequences, and describes the prevalence of food insecurity in four categories—food secure, mildly food insecure, moderately food insecure and severely food insecure as described in Table 1.

**Table 1:** Constituents of household food insecurity categories.

Household food insecurity (access) categories	Conditions experienced by household
Food secure	Experiences none of the food insecurity (access) conditions and worry over food, but just rarely.
Mildly food insecure	Worries about not having enough food sometimes or often and/or being unable to eat preferred foods, and/or eats a more monotonous diet and/or some food considered undesirable, but only rarely. But it does not cut back on quality nor experience any of the three most severe conditions (running out of food, going to bed hungry, or going a whole day and night without eating).
Moderately food insecure	Sacrifices quality more frequently, by eating a monotonous diet or undesirable foods sometimes or often, and/or has started to cut back on quantity by reducing the size of meals or number of meals, rarely or sometimes. But it does not experience any of the three most severe conditions.
Severely food insecure	Cuts back on meal size or the number of meals often, and/or experiences any of the three most severe conditions, even as infrequently as rarely.

*Source*: Coates et al. (2007).

The household's score is the total score of each household based on the frequency of occurrence of the household food insecurity conditions as reflected on the questions (3 = often, 2 = sometimes, 1 = rarely) with the lowest score ‘0’ and the highest score ‘27’. The higher the score, the more food insecure and the lower the score the more food secure.

The HDDS is a validated tool (Swindale & Bilinsky, 2006; Vellema et al., 2016) that serves as a dietary indicator or a monitor of food access seasonal fluctuation, it measures household dietary diversity using 12 food groups and involves a 24‐h household dietary recall to adequately reflect the quality of a household diet. Twelve food groups were investigated comprising white roots and tubers, cereals, fruits, fish and seafood, meat, eggs, oils and fats, milk and milk products, sweets, legumes, nuts and seeds and spices and condiments. The maximum score of the HDDS is ‘12’ with each food group scoring ‘1’. The minimum score by a household is ‘0’. The higher the score, the more diverse the household diet, and the lesser the score, the less diverse their diet.

Both surveys have been demonstrated as reliable and valid for rapid data collection and analysis in several countries (Deitchler et al., 2010; Ene‐Obong et al., 2017; Frayne & McCordic, 2015; Gucciardi et al., 2014; INDDEX Project, 2018; Jones et al., 2013; Knueppel et al., 2010; Mohammadi et al., 2012; Nour & Abdalla, 2021; Raihan et al., 2018; Swindale & Bilinsky, 2009; Tuholske et al., 2018; Vellema et al., 2016).

The questionnaire had three sections—HFIAS, HDDS and household demographic information. In the HFIAS section, question 1 reflects anxiety and uncertainty over food, question 2−4 reflects insufficient food quality, and question 5–9 reflects insufficient food intake. The response options include ‘1’ and ‘0’ indicating ‘yes’ and ‘No’ respectively. The nine subquestions probed the frequency of occurrence of the food insecurity situation. The HDDS survey of 12 food groups allowed respondents to indicate if any of the food groups were consumed by their household members within the last 24 h of the investigation. The response code ‘1 and 0’ means ‘yes and no’ respectively. The third section of the questionnaire collected demographic data on household income and size, age, husband and wife's education level, work status, household head and marital status. The data were structured in categories. For instance, the household head was categorised as ‘male and female’; work status was further categorised into ‘farmers, business, those in paid jobs, full‐time housewives and those not working.’ See Table 2 for details. These categories are relevant to characterise household food security further. Research instrument reliability using the Cronbach alpha coefficient (Pallent, 2001) gave HFIAS—0.87, HDDS—0.74, and the household demographics—0.72.

**Table 2:** demographic characteristics.

Items	Frequency	Percentage
*Age of respondents*		
20‐30	90	23.1
31‐40	124	31.8
41‐50	107	27.4
51‐Above	69	17.7
*Marital status of respondents*		
Married	276	70.8
Divorced	27	6.9
Widow	67	17.2
Single parent	20	5.1
*Family head*		
Male	276	70.8
Female	114	29.2
*Family size*		
2‐4	205	52.6
5‐7	137	34.6
8‐10	43	11
11‐above	7	1.8
*Wife's education*		
None	87	22.3
Primary school	94	24.1
Secondary school	34	8.7
Vocational training school	60	15.4
Higher Education	115	29.5
*Husband's education*		
None	67	23.3
Primary school	52	18.1
Secondary school	40	13.9
Vocational training school	40	13.9
Higher Education	89	30.9
*Wife's work status*		
Farmer	157	40.3
Civil servant	141	36.2
Businesswoman	85	21.8
Full‐time housewife	7	1.8
*Husband's work status*		
Farmer	117	40.5
Civil servant	92	31.8
Businessman	72	24.9
No job doing	8	2.8

Household income		
Family income	Husband	Wife
18, 000 – below	49.5%	50.8%
19, 000 – 30, 000	14.7%	12.2%
31, 000 – 50, 000	17.9%	23.4%
51, 000 – 100, 000	7.7%	5.6%
101, 000 – 200, 000	8.4%	6.1%
201, 000 – Above	1.8%	1.9%

### 2.2 Target population and sampling technique

The research population consists of married women, widows and divorcees representing their households within NLGA. They gave information about their husbands and households. The National Population Commission Nigeria (2010) estimated the population of women in Nsukka LGA at 160, 030 and this forms the target population of this study. Household in this study refers to a group of people living in the same house and sharing the same food.

Sample size estimation provided by Yamane (1967) and Glenn (1992) was adopted.

The equation calculates a sample size for any population ≥100,000 at ±5% precision level (*p* = 0.05).

$$n\equiv \frac{N}{1+N{\left(e\right)}^{2}},$$

where *n* represents the sample size and *N* is the population size, while *e* is the level of precision. Therefore,

$$n=\frac{160,030}{1+160,030(.05)(.05)}=400.$$

The formula estimates that a sample of 400 women is appropriate for a survey population of ≥100,000 people. Four hundred women were the target sample size for this study.

### 2.3 Data collection

Survey participants were selected through women's district meetings. These groups made participants' identification and accessibility straightforward. Data collection was conducted between June and August 2019, during the meeting of each district women's group with the consent of the executive members. Participants were recruited through simple random sampling (Fraenkel & Wallen, 2000; Gravetter & Wallnau, 2013). Attendance was taken, and every second person on the attendance list was selected until 25 women were recruited from each group. Anyone that declined was replaced from the list. The participants gave their consent and their right to participation and withdrawal was emphasised. From the 500 questionnaires distributed, 89.6% were returned, 13% were invalid due to multiple answers or incomplete information and were removed during sorting, data entry and cleaning. Three hundred and ninety (87%) questionnaires were valid.

### 2.4 Data analysis

This research addresses the following questions. (1) What is the prevalence of food (in)security in the NLGA? (1b) Is there any difference in the food security of the younger or older women's household; Is there any difference in the food security of households headed by males or females? (2) What factors are associated with food security among the households? (3) What is the household dietary diversity level? To measure the prevalence of household food (in)secure (access) that determined the food (in)security categories and scores, food insecurity access domains and to determine the household dietary diversity level, descriptive statistics such as the measure of central tendency and dispersion, standard deviation, percentages and tables were applied. The Chi‐square test for independence was used to determine the significance of the association between two sets of categorical data, namely the food security categories and demographic factors. To further understand the dynamics of food security within households, the analysis of variance (ANOVA) compared household food security by the age groups of the women and the gender of the household head. SPSS Statistics 21 was used for all the data analysis.

## 3 RESULTS

### 3.1 The demographics

Table 2 presents household characteristics including the gender of the household head, respondent's age, household size, marital status and household income. Women in middle age accounted for 31.8% of the study population, about 70.8% were married, and 70.8% of the households had males as their household heads. Above half (52.6%) of the population had a family size of 2–4. Most of the participants were farmers (40.3%), and nearly in equal proportion with their husbands (40.5%). Most households (55.4%) had two‐three income sources. However, most participants earned N18,000 ($43.4) or below.

### 3.2 The prevalence of food insecurity (access) in Nsukka

The prevalence of food insecurity among households in Nsukka LGA indicated 17.4% of households as food secure and 82.6% of households as food insecure in varying degrees demonstrating an overall high rate of household food insecurity as presented in Table 3.

**Table 3:** The prevalence of food insecurity (access).

Occurrence questions	Degree of food insecurity			
	No (%) 0	Rarely (%) 1	Sometimes (%) 2	Often (%) 3
Worry about food			13.3%	
Unable to eat preferred food			Mildly food insecure	
Eat limited variety of food				
Eat unwanted food				
Eat smaller food			9%	
Eat fewer meals daily	17.4% Food secure		Moderately food insecure	
Have no food of any kind				
Go to sleep hungry		60.3%		
Go a whole day and night without food		Severely food insecure		

### 3.3 The household food insecurity (access‐related domains—food anxiety, food quality and food intake)

The description of the HFI was obtained from the aggregate of the response score to each of the HFIAS occurrence questions. Approximately 72.3% of households experienced anxiety and uncertainty over household food, while 27.7% were not worried or uncertain. Over half of the sampled population experienced insufficient food quality. They either ate unwanted food (65.9%), limited variety of food (63.1%), or unpreferred food (64.6%) due to lack of resources, and less than 40% of households experienced none of these conditions (34.1%, 36.9% and 35.4% respectively). Some households experienced insufficient food intake by going a whole day without food (38.2%), go to sleep hungry (45.1%) or have no food of any kind (49%). More than half ate fewer meals a day (63.3%), and smaller portions of meals (66.2%) due to food inadequacy. The reverse was the case with households who do not experience these conditions (61.8%, 54.9%, 51%, 36.7% and 33.8% respectively).

### 3.4 Food security difference between households of women in different age groups

Table 4 is the descriptive statistics of households' food security scores of women in different age groups. These were further compared using ANOVA. The results revealed a significant mean difference (*p* = 0.001, *p* < 0.05) in the food security score between some age groups, *F* (3, 386) = 6.113, *p* < 0.05, *η* ^2^ = 0.45. Pairwise comparisons revealed that households with women aged 20–30 years had a significantly higher mean HFIAS than those of women aged 41–50 years (*p* = 0.001) and ≥51 years (*p* = 0.004). Conversely, no significant difference (*p* > 0.05) exists between households with women aged 20–30 years and 31–40 years or between women aged 31–40 and 41–50 or ≥51 years. The *R* ^2^ of 0.45 = 45% indicates that age explains 45% of the variation in household food security between groups. The standard deviation of households with women aged 20–30 years was smaller showing consistency in their responses, in contrast to other groups where there was a great range of scores.

**Table 4:** Descriptive statistics of household food security by women's age.

Household Food Insecurity Access (HFIA) score by household						
Age	Mean	Std. Deviation	*N*	Minimum	Maximum	Median
20−30	12.64	6.082	90	0	27	13.00
31−40	10.48	6.946	124	0	27	11.00
41−50	8.54	6.661	107	0	24	9.00
51−Above	9.00	8.403	69	0	27	9.00
Total	10.18	7.111	390	0	27	11.00

### 3.5 Household food security difference between the gender of household heads

Descriptive statistical analysis revealed close median, mean scores and standard deviation for male and female headed households as shown in Table 5. ANOVA revealed no significant difference (*p* = 0.428, *p* > 0.05) between the mean scores for both groups, *F* (1, 388) = 0.630, *p* = 0.428, *η* ^2^ = 0.002. This suggests that the gender of the household heads does not significantly influence household food security.

**Table 5:** Mean of household food security by the gender of household heads.

**Household Food Insecurity Access (HFIA) score by household**						
Family head	Mean	Std. Deviation	*N*	Minimum	Maximum	Median
Man	10.03	7.103	276	0	27	11.00
Woman	10.57	7.148	114	0	27	10.50
Total	10.18	7.111	390	0	27	11.00

### 3.6 Factors associated with household food security

Using the chi‐square test, the demographic factors age of the women, gender of household head, marital status, education, family income, work status and household size were tested for associations with household food security. The chi‐square result in Table 6 revealed a significant association between household food security and age, education, income level and work status. However, no association was found between household food security and household size, gender of household head and marital status.

**Table 6:** Factors associated with food security.

Factors	Sig. level (0.05) Pearson chi‐square	*p*‐value	Outcome
Age of the woman	*X* ^2^ (9, *N* = 390) = 34.5, *p* < 0.05	0.001	Factor
Women's education	*X* ^2^ (12, *N* = 390) = 75.7, *p* < 0.05	0.001	Factor
Husband's education level	*X* ^2^ (12, *N* = 390) = 59.9, *p* < 0.05	0.001	Factor
Wife's work status	*X* ^2^ (9, *N* = 390) = 36.2, *p* < 0.05	0.001	Factor
Husband's work status	*X* ^2^ (9, *N* = 390) = 28.2, *p* < 0.05	0.001	Factor
Wife's income	*X* ^2^ (15, *N* = 390) = 70.4, *p* < 0.05	0.001	Factor
Husband's income	*X* ^2^ (15, *N* = 390) = 94.1, *p* < 0.05	0.001	Factor
Household size	*X* ^2^ (9, *N* = 390) = 16.7, *p* > 0.05	0.053	Not a factor
Gender of household head	*X* ^2^ (3, *N* = 390) = 3.24, *p* > 0.05	0.355	Not a factor
Marital status	*X* ^2^ (9, *N* = 390) = 14.7, *p* > 0.05	0.098	Not a factor

### 3.7 Household dietary diversity

**Figure 1:**
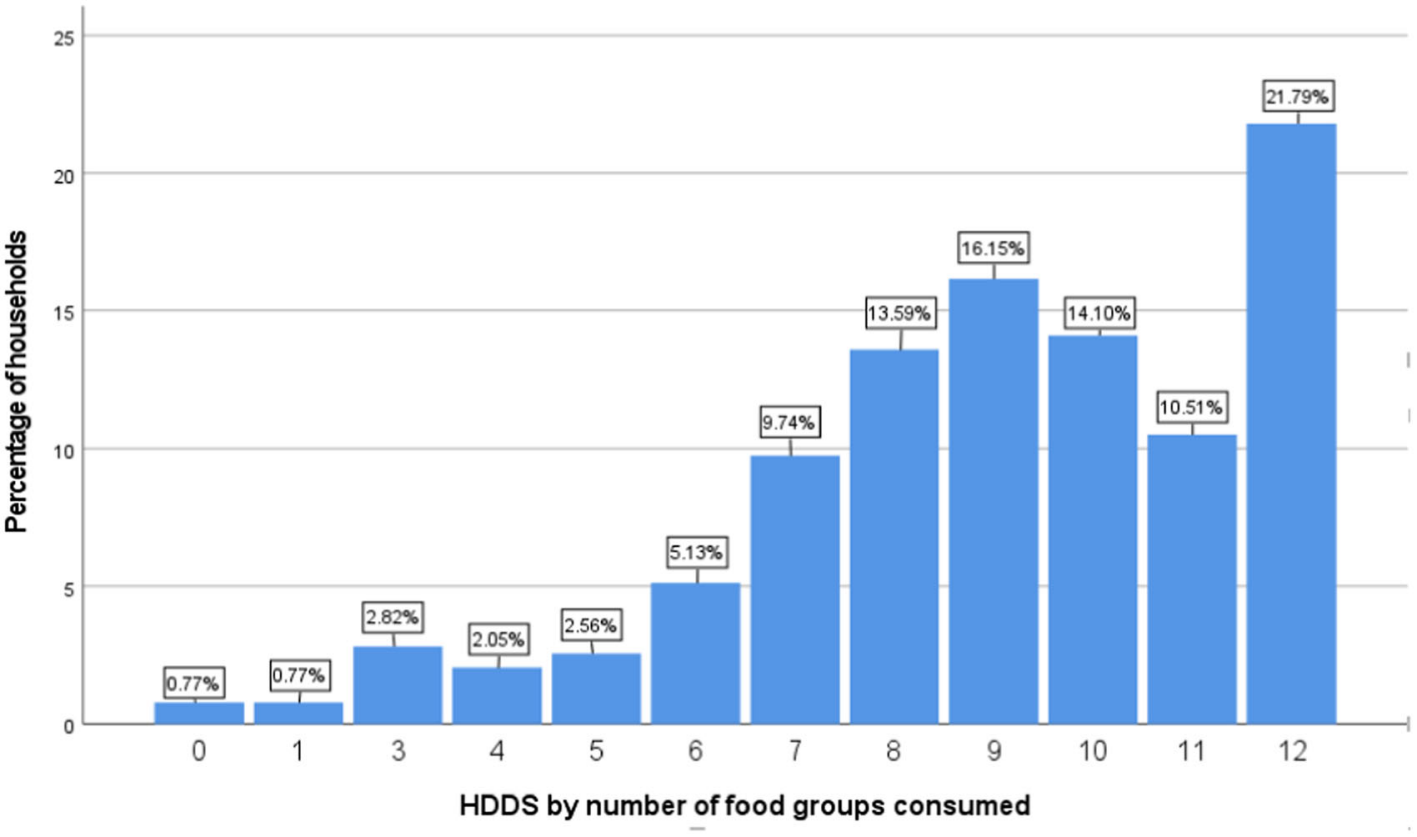
Household dietary diversity score.

The dietary diversity scores of households shown in Figure 1 indicate the level of consumption of the measured food groups. About 75.3% of households headed by males consumed 12 food groups. Approximately 53.6% of households fell at or below the HDDS average score of 9.02 and consumed ≤9 food groups. Males headed 48% of these households, while females headed 64%.

Furthermore, foods mostly consumed by Nsukka households include cereals, consumed at 94.6%, white roots, and tubers at 87.7%, vegetables at 85.9%, and oil and fat at 83.6%. Milk and milk products and eggs, were the least consumed food groups with 42.3% and 40% respectively.

## 4 DISCUSSION

Our research investigated household food security and dietary diversity in NLGA, Southeast Nigeria. Findings revealed high level of food insecurity at 82.6% corresponding with several household food security studies in some rural areas in Nigeria (Arene & Anyaeji, 2010; Ene‐Obong et al., 2017; Nour & Abdalla, 2021; Obayelu, 2012; Olawuyi, 2019; Olayinka et al., 2016; Omotesho et al., 2006; Tsegaye et al., 2018). Similar situations were reported in some parts of Africa. Knueppel et al. (2010) found 79.3% of household food insecurity in rural Tanzania. Crush et al. (2012) reported a significant level of food insecurity in South Africa, Zimbabwe and Swaziland, and 70% of household food insecurity was reported in Ghana (Tuholske et al., 2018). Lack of food is a consequence of the unavailability of food or households' inability to access the available food. Food inaccessibility is a major issue within Nsukka households, meaning that most households lack the resources to access food. For instance, the survey revealed a low household monthly food income, about half of adult male and female household members earned approximately N18,000 ($43.4) or below/per month (see Table 2). Frayne and McCordic (2015) found income as a strong predictor of household food security, indicating that high household income could suggest household food security. The high level of food insecurity affirms the high level (60%) of poverty recorded in NLGA (Ali & Agbiogwu, 2014) and significant level of malnutrition among women and children (Nzeagwu & Aleke, 2016). Furthermore, the location of University of Nigeria in Nsukka, may have influenced the educational attainment of the population, but 46.4% and 41.1% of the respondent and their husbands respectively had primary or no education. This could impact their level of awareness of food, nutrition and security, knowledge, technique and approach to farming and nonfarming income initiative and activities. Ifeoma and Agwu (2014) agreed that education can enhance the knowledge of innovative farming and positively inform decisions on household food production and nutrition.

The experience of anxiety and uncertainty was common across the research population. Nearly three‐quarters of the households were worried and uncertain about their next meal. This is congruent with Saaka et al. (2017) who reported that 60% of households in Northern Ghana worry about food insufficiency. Research shows that food insecurity causes a considerable burden of anxiety and depression (Bukhaman et al., 2020). Johnson (2021) concluded that the state of anxiety, stress, and stigma due to food insecurity can contribute to poor dietary intake, poor mental health and the development of chronic diseases, particularly in women. Congruently, Hadley and Patil (2008) found food insecurity as a strong predictor of depression and anxiety symptoms. This was echoed by Arenas et al. (2019), Fang et al. (2021) and Melchior et al. (2012).

Food security exists when people have access to their preferred food, making choice an important factor in food security (Food Agricultural Organisation, 1996). However, this was not the case with Nsukka households, more than half of the households either ate unwanted food, limited variety of food, or unpreferred food. This is consistent with Saaka et al. (2017) who reported a 60% and 63% consumption of unwanted food and unpreferred food respectively in Northern Ghana.

Some households confirmed going a whole day without food (38.2%) or sleeping at night hungry (45.1%). More households ate fewer meals and smaller portions of food in a day. This result concurs with Saaka et al. (2017), Tsegaye et al. (2018). Food compromise is usually the initial response to food insufficiency. At first, food quality is reduced, and then food quantity as the situation worsens. Households could reduce food quality by consuming unwanted food, a limited variety of food, or unpreferred food (Olajide & Doppler, 2013). As the situation deteriorates, households are more likely to consume smaller portions of food and eat fewer meals a day. Further cuts on meal frequency results in sleeping at night without food and going hungry a whole day. The implication of food quality and quantity compromise raises questions that need further investigation around family nutrition, especially, in children, women of reproductive age, the elderly, and other immunocompromised groups. Poor quality food may not provide the recommended dietary allowance the body needs to function adequately for a healthy life (Webb et al., 2018). Poor nutrition depresses the body's immunity and upsurges susceptibility to diseases and infections (Keusch, 2003; Shetty, 2010)

Furthermore, comparing Food security difference between households of women in different age groups showed a significant variation. The food security level of households with women aged 20–30 years varied significantly with a moderate effect (*R* ^2^ = 0.45) when compared to households with women aged 41–50 years and above. Households with younger women appear more food insecure when compared to households with older women. Yet, households with younger women had smaller family sizes, and could be more proactive, enlightened, receptive, and self‐motivated towards household food security, diet and nutrition. However, older women could be more experienced and knowledgeable in household food nutrition than younger women. The older women in the sample had more access than the younger women. They earned more (57.2%) and had children who earned higher (64.3%). Their households had more than three sources of income (51.3%).

Further comparison between the gender of household heads revealed no significant difference in their food security levels. This suggests that the gender of the household head may not significantly influence household food security. Previously published literature on the relationship between the gender of the household head and household food security is controversial, with some reports suggesting that female‐headed households are more likely to be food secure than male (Adepoju & Adejare, 2013) and some reporting the reverse due to gender inequalities, low education, low employment and other factors affecting women in society (Felker‐Kantor & Wood, 2012; Matemilola & Elegbede, 2017; Nwaka et al., 2020). Women's access to education and paid jobs may explain the comparable levels of food security between male and female‐headed households in this study sample. More than half of the women had some form of formal education with the majority at the basic level. This could be partly attributed to the presence of one of the first and most renowned universities in Nigeria. Nzeagwu and Aleke (2016) echoed the University of Nigeria Nsukka's positive influence on women's education and invariably on their food security level.

Chi‐square test for factors that are associated with household food security. revealed that the age of the women, husband and wife's education levels, work status and income level were associated with household food security. This result is consistent with Obayelu (2012), Abu and Soom (2016) who found a positive relationship between education, income and household food security. While Adepoju and Adejare (2013) found determinants of food insecurity to include the gender and education of the household head. No significant association was found between food security level and household size, gender of household heads, and marital status. These factors did not influence the household food security of the sampled population. This is congruent with Abu and Soom (2016) who found a negative relationship between household size and household food security. However, in contrast, Adepoju and Adejare (2013) concluded that household size and the gender of the household head are determinants of household food insecurity.

We investigated household dietary diversity which explains the different food groups consumed by the sampled population. Less than one‐quarter of the households consumed all the food groups, and more than half (53.6%) fell at or below the average dietary diversity score of the group, constituting more of female headed households. The most consumed food groups in descending order were cereals, white roots, tubers, vegetables and oils/fats. These are the major crops farmed by households in the region and they form the basis for most family menus. This corresponds with Nzeagwu and Aleke (2016) who found that in one of the NLGA communities, many households had several food crops, especially roots and tubers but lacked the financial power to acquire enough food. The high consumption of cereals, tubers, and roots, which are staples in the region (Nduka & Ozioma, 2019), explains why most households cultivate them. Also, they are affordable and energy‐dense, enough to satisfy and shield hunger for a longer period. They are cultivated and traditionally valued in the southeast region (Ogbonna et al., 2012). For instance, the region celebrates the new yam festival during the yam harvest season (Obidiegwu & Akpabio, 2017). However, the importance of the recommended amount of protein, vitamins and minerals in a diet cannot be overlooked. Henjum et al. (2015) found that low dietary diversity explains the low intake of micronutrient. Rich protein foods like milk and milk products and eggs were the least consumed food groups. This finding corresponds to Ene‐Obong et al. (2017) who discovered that in some parts of the Southeast, eggs, and milk were not consumed frequently, in fact, they are not prominent in the Igbo cultural food system. They are referred to as ‘privileged food’ owing to their high costs. Again, this calls for further research as it raises a question of nutrition especially, among vulnerable groups.

In conclusion, the high level of anxiety and uncertainty over food and the poor intake of protein‐rich foods raises concerns about the physical and psychological health and nutritional status of household members. Government–private partnership interventions are important to alleviating food insecurity within the area while locals are equipped for better productivity towards achieving Sustainable Development Goals.

## AUTHOR CONTRIBUTIONS

Ijeoma C. Ukonu, Carol A. Wallace and Nicola M. Lowe substantially contributed to the conception and design of the study. Ijeoma C. Ukonu led the collection of data and performance of the data analysis and the draft of the manuscript. Carol A. Wallace and Nicola M. Lowe revised the manuscript critically for important intellectual content.

## CONFLICT OF INTEREST STATEMENT

The authors declare no conflict of interest.

## ETHICS STATEMENT

Ethical approval for the study was granted by the University of Central Lancashire (UCLAN), UCLAN STEMH (Science, Technology, Engineering, Medicine, and Health) ethics committee and permission was obtained from the Nsukka LGA Women's Organisation. Participants' confidentiality was achieved such that participants could not be identified by the researchers from their questionnaire responses once they had been completed (Poldrugovac et al., 2016).

## Data Availability

The data that support the findings of this study are available on request from the corresponding author. The data are not publicly available due to privacy or ethical restrictions.
